# Long-term study of *Borrelia* and *Babesia* prevalence and co-infection in *Ixodes ricinus* and *Dermacentor recticulatus* ticks removed from humans in Poland, 2016–2019

**DOI:** 10.1186/s13071-021-04849-5

**Published:** 2021-07-01

**Authors:** Agnieszka Pawełczyk, Małgorzata Bednarska, Adrianna Hamera, Emilia Religa, Milena Poryszewska, Ewa J. Mierzejewska, Renata Welc-Falęciak

**Affiliations:** 1 Department of Immunopathology of Infectious and Parasitic Diseases, Medical University of Warsaw, 3C Pawińskiego Street, 02-106 Warsaw, Poland; 2 Department of Parasitology, Faculty of Biology, University of Warsaw, 1 Miecznikowa Street, 02-096 Warsaw, Poland; 3 Wild Urban Evolution and Ecology Lab, Centre of New Technologies, Banacha 2c Street, 02-097 Warsaw, Poland

**Keywords:** Lyme borreliosis, Babesiosis, Co-infection, Ticks, Tick-borne diseases, *Borrelia*, *Babesia*

## Abstract

**Background:**

Lyme borreliosis (LB) is the most common vector-borne disease in Europe. Monitoring changes in the prevalence of different *Borrelia* species in ticks may be an important indicator of risk assessment and of differences in pathogenicity in humans. The objective of our study was to assess the prevalence, co-infection and distribution of *Borrelia* and *Babesia* species in ticks removed from humans in a large sample collected during a study period of 4 years.

**Methods:**

The ticks were collected throughout Poland from March to November over 4-year period from 2016 to 2019. All ticks (*n* = 1953) were morphologically identified in terms of species and developmental stage. Molecular screening for *Borrelia* and *Babesia* by amplification of the flagellin gene (*flaB*) or* 18S* rRNA marker was performed. Pathogen identity was confirmed by Sanger sequencing or PCR–restriction fragment length polymorphism analysis.

**Results:**

The ticks removed from humans in Poland during this study belonged to two species: *Ixodes ricinus* (97%) and *Dermacentor reticulatus* (3%). High *Borrelia* prevalence (25.3%), including *B. miyamotoi* (8.4%), was confirmed in *Ixodes ricinus* ticks removed from humans, as was the change in frequency of occurrence of *Borrelia* species during the 4-year study. Despite *Babesia* prevalence being relatively low (1.3%), the majority of tested isolates are considered to be pathogenic to humans. *Babesia* infection was observed more frequently among *Borrelia*-positive ticks (2.7%) than among ticks uninfected with *Borrelia* (0.8%). The most frequent dual co-infections were between *Borrelia afzelii* and *Babesia microti.* The presence of *Borrelia* was also confirmed in *D. reticulatus* (12.7%); however the role of these ticks in spirochete transmission to susceptible hosts is still unclear.

**Conclusions:**

Although the overall risk of developing LB after a tick bite is low in Europe, knowledge of the prevalence and distribution of *Borrelia* and *Babesia* species in ticks might be an important indicator of the risk of both these tick-borne diseases.

**Graphical abstract:**

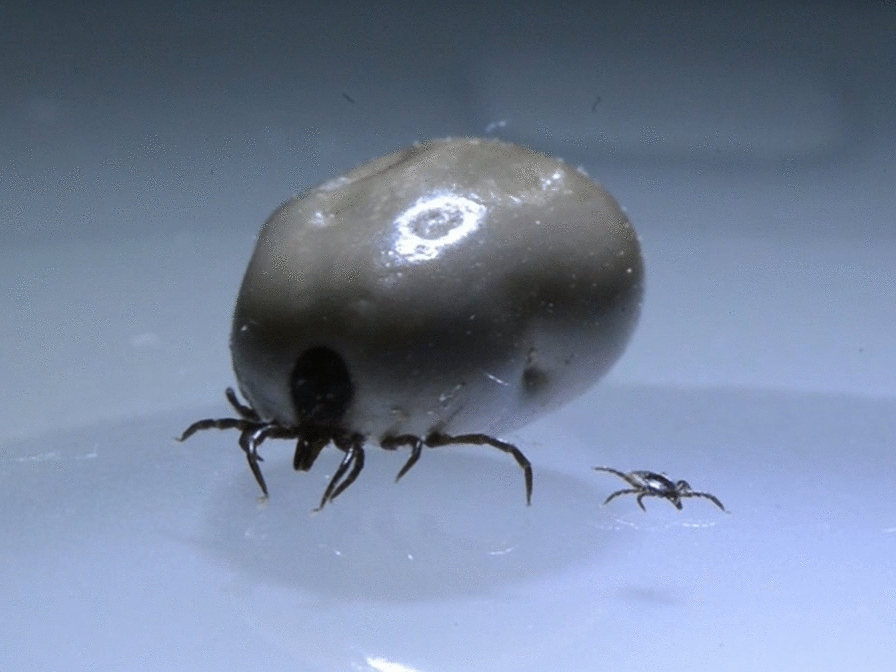

**Supplementary Information:**

The online version contains supplementary material available at 10.1186/s13071-021-04849-5.

## Background

With 85,000 cases reported annually, Lyme borreliosis (LB) is the most common vector-borne disease in Europe [1]. The estimated incidence of LB in Poland increased dramatically from 20.3 per 100,000 inhabitants in 2007 to 53.6 per 100,000 inhabitants in 2019 (an estimated average increase from 7735 cases per year in 2007 to 20,614 cases per year in 2019) [2]. However, the reliability of current incidence data on LB is uncertain due to diagnostic problems and limited reporting [3]. At least five species of *Borrelia*, namely *Borrelia burgdorferi* (*s.s.*), *B. garinii*, *B. afzelii*, *B. spielmanii* and *B. bavariensis*, are known to be pathogenic to humans, with each of these species believed to be associated with different clinical manifestations. The heterogeneity among *B. burgdorferi* (*s.l.*) species seems to be the main factor causing the regional differences in the clinical expression of human LB [4]. *Borrelia burgdorferi* (*s.s.*) is believed to be arthritogenic, *B. afzelii* causes mainly skin infections and *B. garinii* is especially neurotropic. Recently, *Borrelia miyamotoi* has been identified as a human pathogen related to relapsing fever in Europe [5] and little is known about its local impact on human health. *Borrelia miyamotoi* disease (BMD) has also been confirmed in an immunocompetent patient, and BMD concurrent with Lyme disease has also been described [6].

In Europe, cases of human babesiosis is reported sporadically, with about 60 confirmed cases caused mainly by *Babesia divergens* described to date in several European countries, including France, UK, Austria, the Czech Republic, Finland, Germany, Italy, Portugal, Switzerland and Poland (for review, see [7]). Non-specific clinical symptoms of human babesiosis, such as fever, flu-like symptoms, headaches, chills, sweats and myalgia, as well as the diagnostic difficulties have a key impact on their correct diagnosis and, consequently, effective treatment [8]. *Babesia microti* infections in immunocompetent individuals often have an asymptomatic but chronic course [9]. In terms of safe blood donation, this is of fundamental importance, especially if blood recipients are immunosuppressed. Transfusion-transmitted babesiosis is being increasingly described globally, mainly in the USA [10].

The tick species *Ixodes ricinus* is associated with deciduous and mixed forests, but the increasing numbers of *I. ricinus* observed over the past decades have allowed it to extend its range to northern areas of the continent and areas located at a higher altitude [11]. Cull et al. [12] have shown that *I. ricinus* typically constitute 90–100% of all ticks removed from humans in Europe and that nymphs are the most commonly detected life stage associated with zoonotic pathogen transmission. The increase in the density of ticks, also in urban areas, and the prolonged period of activity of these arachnids are probably the result of changes occurring in the environment, such as in land use in agriculture, forest management, changes in abundance and distribution of free living animals and climate change [13–16]. The observed phenomena translate directly into an increase in the risk of transmission of pathogens vectored by ticks, which can be a significant health problem, especially for people with an impaired immune system, whose percentage in the general population is steadily increasing [17].

*Ixodes ricinus* ticks are competent vectors for many species of pathogenic viruses, bacteria and protozoa. An important problem in the epidemiology of tick-borne diseases is co-infection, i.e. simultaneous, multi-species infections; such infections are especially difficult to diagnose in humans [18]. Co-infection in humans and animals might enhance disease severity and may have significant consequences in terms of tick-borne disease treatment and diagnosis [18, 19].

Knowledge of *Borrelia* prevalence and species distribution is crucial to our understanding of the epidemiology as well as of the prevention and diagnosis of LB. There are only a limited number of studies on the prevalence of specific pathogenic species in ticks removed from humans, which mainly provided information on the *B. burgdorferi* (*s.l.*) complex. In Poland, most of the studies conducted to date were on questing ticks or ticks collected from animals [20–27]. However, the evaluation of pathogens in ticks parasitizing humans may provide specific information on the risk of human exposure to tick-borne infections. The aim of our study was to assess the prevalence and distribution of *Borrelia* and *Babesia* species in ticks removed from humans in Poland, in a large sample collected during a 4-year study period.

## Methods

### Tick collection and identification

The research reported here was conducted over 4-year period from 2016 to 2019. The ticks were collected throughout Poland from March to November of each year and then were delivered directly by patients or by a delivery company in a tightly-sealed, ethanol-filled container to the Diagnostic Laboratory of Parasitic Diseases and Zoonotic Infections AmerLab Ltd, a spin-off company of the University of Warsaw and Medical University of Warsaw, within 5 days after removal from the skin by a physician or the patients themselves. Ticks were morphologically identified in terms of species and developmental stage using a standard taxonomic key [28]. Specimens that could not be identified due to being extensively damaged when being removed from the skin were not included in the study.

### DNA extraction and PCR analysis

Individual larvae, nymph and adult ticks were washed in sterile ethanol and then in sterile water to avoid DNA contamination and then homogenized using sterile a mini-homogenizer with hand-held mixing motor drive (EURx, Gdańsk, Poland). Genomic DNA from ticks was isolated using the DNeasy Blood & Tissue Kit (Qiagen, Hilden, Germany) according to the manufacturer’s protocol. Genomic DNA was used for molecular screening for spirochetes through amplification of the flagellin gene (*flaB*) marker, using the primers reported in [29]. Initial PCR conditions were modified as follows: 95 °C, 5 min (initial denaturation); then 95 °C/30 s (denaturation), 52 °C/30 s (primer annealing), 72 °C/80 s (elongation), for 35 cycles; and a final elongation at 72 °C for 7 min. Nested PCR was performed with minor modification: 95 °C, 20 s (denaturation); then 55 °C/20 s (denaturation), 72 °C/60 s (elongation). For *B. miyamotoi* detection among positive samples, specific primers for the* flaB* marker were used [24]. *Babesia* spp. were detected and identified using GR2 and GF2 primers targeting the* 18S* rDNA fragment. The primers and thermal profiles used in this study are described in [30]. Negative controls were performed in the absence of template DNA. *Babesia microti* King’s College strain DNA isolated from infected BALB/c mice blood and sequenced *Borrelia* DNA obtained from infected ticks [24] were used as positive controls. PCR products were visualized in 1.5% agarose gels stained with Midori Green Stain (Nippon Genetics Europe, Düren, Germany).

### Identification of *Borrelia* and *Babesia* species

*Borrelia*-positive samples from ticks collected in 2016 and 2017 and *Babesia*-positive samples from ticks collected in 2016, 2017 and 2018 were sequenced by a private company (Genomed S.A., Warszawa, Poland) in both directions. Obtained nucleotide sequences were analyzed using BLAST NCBI and MEGA v. 7.0 software [31] for sequence alignment and species typing using sequences deposited in GenBank NCBI. The new nucleotide sequences have been deposited in the GenBank database under accession numbers MW791411–MW791420.

Restriction fragment length polymorphism (RFLP) was used to differentiate *Borrelia*-positive isolates at the species level obtained in 2018 and 2019. Positive amplicons after nested PCR were digested with the restriction enzyme HpyF3I (Thermo Fisher Scientific, Waltham, MA USA), which recognizes the 5′C↓TNAG3′ sequence [29], according to the manufacturer’s protocol. The digestion products were separated in a 2% agarose gel, visualized and archived in the GelDoc-It imaging system (Thermo Fisher Scientific). The obtained restriction patterns enabled recognition of species of the *B. burgdorferi* complex and *B. miyamotoi*.

### Statistical analysis

Statistical analysis was performed using IBM SPSS Statistics v. 25.0 software (IBM Corp., Armonk, NY, USA). Prevalence of *Borrelia* and *Babesia* infection (percentage of ticks infected) was analyzed using maximum likelihood techniques based on log-linear analysis of contingency tables (HILOGLINEAR). For analysis of the prevalence of *Borrelia* and *Babesia* in ticks, we fitted the prevalence of pathogens as a binary factor (infected = 1, uninfected = 0) and then by year (4 levels: 2016–2019 for *Borrelia*; 3 levels: 2016–2018 for *Babesia*) and tick stadium (larvae, nymphs, adults). *P* values < 0.05 were considered to be statistically significant.

## Results

### *Ixodes ricinus* and *D. reticulatus* ticks

Almost all ticks collected from humans in during the study period (2016–2019) were *I. ricinus* (97%), with the other tick species identified being *D. reticulatus* (3%).

**Fig. 1:**
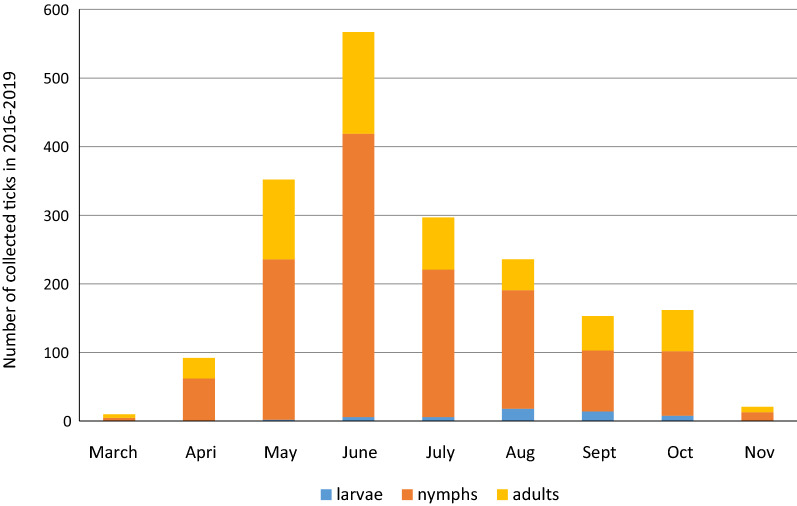
Total number of *Ixodes ricinus* ticks by stage and month collected during the 4 years of the study. Statistical differences between tick stages (larvae, nymphs, adults) and month of study (March–November): *P* < 0.000

A total of 1890 *I. ricinus* ticks were collected from humans during the study period, of which 54 (2.9%) were larvae, 1298 (68.7%) were nymphs, 524 (27.7%) were females and 14 (0.7%) were males. Most of these ticks were collected in 2018–2019 (*n* = 762 and *n* = 775, respectively), whereas in 2016–2017 only 353 ticks were analyzed (*n* = 126 and *n* = 227, respectively). Two peaks of tick activity were observed: the first in June, followed by a second one in October; however, the mean number of ticks collected in October was almost fourfold lower than that in June (Fig. 1). The number of ticks in each stadium (larvae, nymphs and adults) removed from humans varied significantly between the months of study (month × number of* I. ricinus* ticks in each stadium:* χ*^2^_16_ = 85.5, *P* < 0.000; Fig. 1; Additional File 1). Over the entire study period, the median number of larvae collected per month was six, with a minimum of two larvae (in May), a maximum of 18 larvae (in July) and no larvae collected in March–April and November. The highest number of nymphs (*n* = 413) and adults (*n* = 148) was noted in June, whereas the median number of ticks collected by month was 144 for nymphs and 60 for adults.

**Fig. 2:**
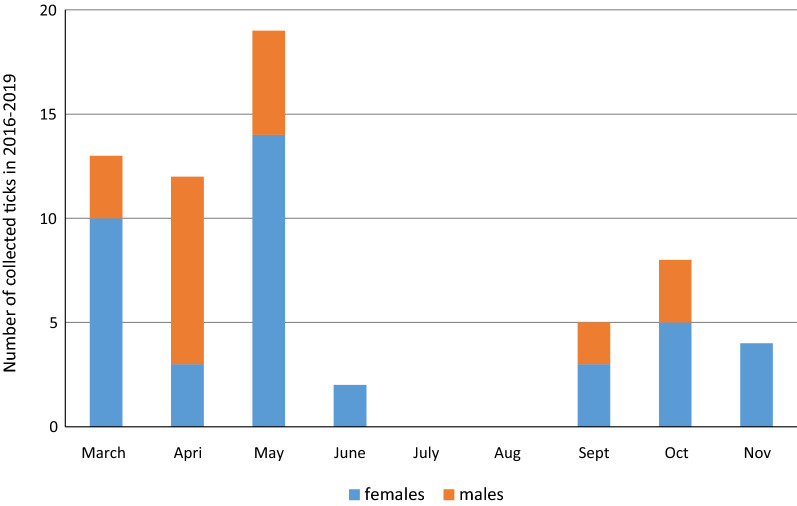
Total number of *Dermacentor reticulatus* ticks by stage and month collected during years of study. Statistical differences between tick stages (females, males) and month of study (March–November): *P* = 0.054

A total of 63 *D. reticulatus* ticks were collected from humans during the study period, of which 41 (65%) were females and 22 (35%) were males. Most *D. reticulatus* ticks were collected in 2018 and 2019 (*n* = 54; 85.7%). Over the entire study period, the median number of ticks collected monthly was seven; however, the highest number of ticks was noted from March to May (21 and 21, respectively; 30% of all collected ticks), and no *D. reticulatus* ticks were observed in July and August (month × number of* D. reticulatus* ticks in each stadium:* χ*^2^_8_ = 14.8; *P* = 0.054) (Fig. 2; Additional File 2).

### *Borrelia* prevalence in *I. ricinus* and *D. reticulatus* ticks

Overall, the prevalence of *Borrelia* infection in the *I. ricinus* ticks removed from humans, as determined by nested PCR, was 25.3% (479/1890; CI: 23.4–27.3%) (Table 1). Statistical analysis of prevalence over the long term (i.e. the entire study period) revealed a significant decrease in *Borrelia* prevalence between 2016 (30.2%; CI: 22.7–38.6%) and 2019 (23.4%; CI: 20.5–26.4%) (*χ*^2^_3_ = 7.58; *P* = 0.051; Table 1). A significant effect of tick stage was also observed (*χ*^2^_2_ = 11.9; *P* = 0.003). *Borrelia* DNA was detected in 9.3% (5/54; CI: 3.6–19.1%) of *Ixodes* larvae, 24.7% (321/1298; CI: 22.5–27.2%) of *Ixodes* nymphs and 28.4% (153/538; CI: 24.7–32.3%) of adult *Ixodes* ticks (Table 1).

**Table 1:** Stadium and year distribution of *Borrelia*-infected ticks removed from humans between 2016 and 2019

Stadium of *Borrelia*-infected ticks	No. of tested ticks (*n*)	No of positive ticks^a^					*P* value
		2016	2017	2018	2019	Total	
*Borrelia*-positive *Ixodes ricinus* ticks^b^							
Larvae	54	2 (28.6; 6.5–64.8)	0 (0.0)	2 (8.7; 1.9–25.1)	1 (7.7; 0.8–30.7)	5 (9.3; 3.6–19.1)	= 0.231
Nymphs	1298	22 (27.5; 18.6–38.0)	38 (29.2; 21.9–37.4)	129 (24.5; 21.0–28.3)	132 (23.6; 20.2–27.2)	321 (24.7; 22.5–27.2)	= 0.550
Adults	538	14 (35.9; 22.3–51.5)	28 (32.6; 23.4–42.9)	63 (29.7; 23.9–36.1)	48 (23.8; 18.3–30.0)	153 (28.4; 24.7–32.3)	= 0.247
Total	1890	38 (30.2; 22.7–38.6)	66 (29.1; 23.5–35.2)	194 (25.5; 22.5–28.6)	181 (23.4; 20.5–26.4)	479 (25.3; 23.4–27.3)	= 0.051*
*Borrelia*-positive *Dermacentor reticulatus* ticks^c^							
Adults	63	1 (20; 2.3–62.9)	1 (25; 2.8–71.6)	2 (7.7; 1.6–22.5)	4 (14.3; 5.0–30.5)	8 (12.7; 6.1–22.2)	= 0.740

*Hierarchic logline analysis: year of study ×  *Borrelia prevalence* in *Ixodes ricinus* ticks: *χ*^2^_3_ = 7.6; *P* = 0.051

^a^Values for positive ticks are given as the percentage with the 95% confidence interval (CI) in parentheses

^b^Hierarchic logline analysis included: year of study ×  *I. ricinus* tick stages (larvae, nymphs, adults) ×  *Borrelia* prevalence: *χ*^2^_6_ = 5.4; *P* = 0.500

^c^Hierarchic logline analysis included: year of study × * D. reticulatus* tick stages (females, males) × * Borrelia* prevalence: *χ*^2^_3_ = 2.2; *P* = 0.542

In total, 12.7% (8/63; CI: 6.1–22.2%) of the *D. reticulatus* ticks delivered to the laboratory during the 2016–2019 study period tested positive for *Borrelia* infections (Table 1). Prevalence of infection decreased from 20% (CI: 2.3–62.9%) in 2016 and 25% (CI: 2.8–71.6%) in 2017 to 7.7% (CI: 1.6–22.5%) in 2018 and 14.3% (CI: 5.0–30.5%) in 2019; however, only nine *D. reticulatus* ticks were tested within the first 2 years of the study (Table 1). Female ticks (9.8%; CI: 3.4–21.5%) were infected less often than males (18.2%; CI: 6.5–37.6%); however the differences between males and females were not statistically significant (*P* = 0.348).

### *Borrelia* species in *I. ricinus and D. reticulatus* ticks

Species typing was conducted on the basis of sequencing of fragments of the *flaB* gene (540-bp product) or RFLP-PCR analysis. Species differentiation of *Borrelia*-infected ticks was successful in 251 of the 479 *Borrelia*-positive tick samples (52.4%) and, according to life stage, in 91 of 153 adults (59%), 157 of 321 nymphs (49%) and three of five larvae (60%). The most frequently detected *Borrelia* species was *B. afzelii* (65.3%; CI: 59.3–71.0%), followed by *B. burgdorferi* (10.8%; CI: 7.4–15.0%)), *B. garinii* (8.8%; CI: 5.7–12.7%), *B. valaisiana* (5.2%; CI: 2.9–8.4%), *B. spielmanii* (1.2%; CI: 0.3–3.2%) and *B. lusitaniae* (0.4%; CI: 0.0–1.8%) (Table 2). The spirochete *B. miyamotoi*, which causes relapsing fever, was identified in 8.4% (CI: 5.4–12.3%) of analyzed ticks.

**Table 2:** *Borrelia* species distribution in infected *I. ricinus* ticks (*n* = 251) removed from humans

*Borrelia* species distribution	No of tested ticks	No of positive ticks)^a, b^						
		*B. afzelii*	*B. garinii*	*B. burgdorferi*	*B. miyamotoi*	*B. valaisiana*	*B. lusitaniae*	*B. spielmanii*
Total	251	164 (65.3; 59.3–71.0)	22 (8.8; 5.7–12.7)	27 (10.8; 7.4–15.0)	21 (8.4; 5.4–12.3)	13 (5.2; 2.9–8.4)	1 (0.4; 0.0–1.8)	3 (1.2; 0.3–3.2)
Tick stage								
Larvae	3	2 (66.7; 17.7–96.1)	0	0	0	0	1 (33.3; 3.9–82.3)	0
Nymphs	157	105 (66.9; 59.3–73.9)	11 (7.0; 3.8–11.8)	18 (11.5; 7.2–17.1)	12 (7.6; 4.2–12.6)	8 (5.1; 2.4–9.4)	0	3 (1.9; 0.5–5.0)
Adults	91	57 (62.6; 52.4–72.1)	11 (12.1; 6.6–19.9)	9 (9.9; 5.0–17.3)	9 (9.9; 5.0–17.3)	5 (5.5; 2.1–11.6)	0	0

^a^Values for positive ticks are given as the percentage with the 95% CI in parentheses

^b^Hierarchic logline analysis included *Borrelia* species prevalence × tick stages (larvae, nymph, adults): *χ*^2^_12_ = 15.2; *P* = 0.231

*Borrelia* species distribution showed no significant differences between tick stages (*P* = 0.231) (Table 2). Adult ticks were more frequently infected with *B. afzelii* (62.6%, 57/91; CI: 52.4–72.1%) and *B. garinii* (12.1%, 11/91; CI: 6.6–19.9%) than with other *Borrelia* species . In nymphs, the most commonly detected species were *B. afzelii* (66.9%, 105/157; CI: 59.3–73.9%) and *B. burgdorferi* (11.5%, 18/157; CI: 7.2–17.1%). Larvae were infected only with *B. afzelii* (66.7%, 2/3; CI: 17.7–96.1%) and *B. lusitaniae* (33.3%, 1/3; CI: 3.9–82.3%).

**Fig. 3:**
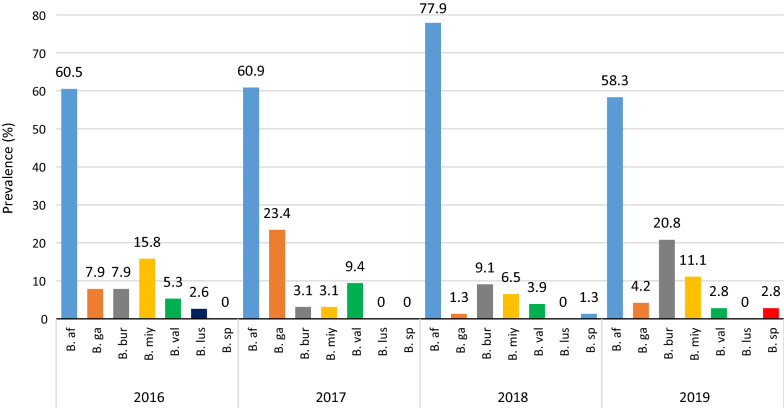
Prevalence of *Borrelia burgdorferi* species in *I. ricinus* ticks removed from humans according to study year (2016–2019). *B. af Borrelia afzelii*,* B. ga B. garinii*,* B. bur B. burgdorferi*,* B. miy B. miyamotoi*,* B. val* *B. valaisiana*,* B. lus B. lusitaniae*,* B. sp B. spielmanii*. Hierarchic logline analysis: year of study × *Borrelia* species prevalence: *χ*^2^_18_ = 49.9; *P* < 0.000

Species distribution in different sampling years is shown in Fig. 3 (*χ*^2^_18_ = 49.9; *P* < 0.000; Additional File 3). Throughout our 4-year study, the collected ticks were predominantly infected with *B. afzelii* [60.5 (CI: 44.7–74.8%), 60.9 (CI: 48.7–72.2%), 77.9 (CI: 67.7–86.1%) and 58.3% (CI: 46.8–69.2%) in 2016, 2017, 2018 and 2019, respectively]. In 2016, *B. miyamotoi* was detected in 15.8% (CI: 6.9–29.7%) of ticks; in 2017 and 2018 the number of *B. miyamotoi*-infected ticks decreased to 3.1 (CI: 0.7–9.6%) and 6.5%, (CI: 2.5–13.6%) respectively; this was followed by an increase to 11.1% (CI: 5.4–19.9%) in 2019. *Borrelia garinii* was the second most frequently noted species in 2017 (23.4%; CI: 14.4–34.8%); however, only 1.3 (CI: 0.1–5.9%) and 4.2% (CI: 1.2–10.7%) ticks were infected in 2018 and 2019. *Borrelia burgdorferi* was the most frequently identified species after *B. afzelii* in 2018 and 2019 [9.1 (CI: 4.2–17.0%) and 20.8% (CI: 12.7–31.2%)]—despite only 3.1% (CI: 0.7–9.6%) of ticks being infected in 2017.

Analysis of co-infection in multiple infected ticks was performed only using RFLP-PCR in 2018 and 2019. Overall, 2.0% (3/149) of the analyzed *I. ricinus* ticks (two nymphs and female) carried two *Borrelia* species (*B. afzelii* with *B. burgdorferi*/*B. miyamotoi*/*B. spielmanii*), while triple infections were observed in only one (0.7%) female (*B. afzelii*/*B. burgdorferi*/*B. lusitaniae*) (Table 3).

**Table 3:** Co-infection in *I. ricinus* ticks removed from humans between 2016 and 2019

Tick stage	No. of specimens	Pathogen species
Female	1	*Borellia afzelii* + *B. spielmanii*
Nymph	1	*Borrelia afzelii* + *B. burdorferi* (*s.s.*)
Nymph	1	*Borrelia afzelii* + *B. miyamotoi*
Female	1	*Borrelia afzelii* + *B. burgdorferii* (*s.s.*)*.* + *B. lusitaniae*
Female	1	*Borrelia valaisiana* + *Babesia microti*
Nymph Female	2 1	*Borrelia afzelii* + *Babesia microti*
Female	1	*Borrelia afzelii* + *Babesia canis*
Female	2	*Borrelia afzelii* + *Babesia venatorum*

**Fig. 4:**
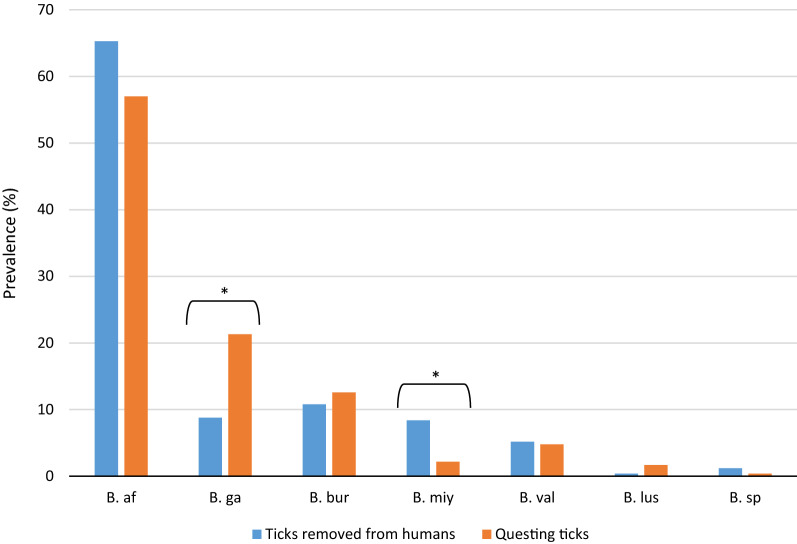
Comparison of *Borrelia* species prevalence in *I. ricinus* ticks removed from humans between 2016 and 2019 (this study) and from questing ticks collected in our previous study [23]. Asterisks indicate statistically significant differences (*P* ≤ 0.05). See caption to Fig. 3 for species names

Comparison of species distribution in *I. ricinus* ticks removed from humans with those from questing ticks in our previous study [24] showed that engorged ticks were by far more frequently infected with *B. myiamotoi* (*P* = 0.003), whereas questing ticks were more commonly infected with *B. garinii* (*P* = 0.0001). These results are shown in detail in Fig. 4.

Species differentiation of *Borrelia*-infected *D. reticulatus* ticks was successful in six of eight *Borrelia*-positive tick samples (75%). All *Borrelia* isolates were identified on the basis of RFLP-PCR analysis, as for *B. afzelii*.

### *Babesia* prevalence in collected ticks

In total, 1.3% (15/1115; CI: 0.8–2.2%) of the *I. ricinus* ticks delivered to the laboratory during the study period tested positive for *Babesia* infections. No significant statistical differences between sex and stage of ticks and year of study were detected. The prevalence of *Babesia* infection ranged from 0.9% (2/227; CI: 0.2–2.8%) in 2017 to 2.4% (3/126; CI: 0.7–6.2%) in 2016. The higher *Babesia* prevalence of 2.4% (8/337; CI: 1.1–4.4%) was found in adult *I. ricinus* comparied to nymphs (0.9%, 7/737; CI: 0.4–1.9%); no infected larvae were noted (Additional File 4).

Species typing was performed on the basis of sequencing of* 18S* rRNA gene fragment (530-bp product); all positive PCR samples were sequenced. Alignment and BLAST-NCBI analyses revealed the presence of three *Babesia* species. Of the 15 isolates, nine (60%) showed a high similarity (> 99%) to the *B. microti* strain Jena isolated originally from a human patient in Germany (EF413181 [32]). The nucleotide sequences of five isolates (33.3%) were identical to *B. venatorum* isolated from human patients in Italy and Austria (AY046575 [33]). One isolate was identified as *B. canis* with a similarity level of > 99% to another Polish isolate (KT272401 [34]).

During 3 years of study (2016, 2017, 2018), one *D. reticulatus* tick (1/36, 2.8%) was infected with *B. canis* which showed a similarity level of > 99% to another Polish isolate (KT272401; [34]).

### *Borrelia* and *Babesia* co-infection in *I. ricinus* ticks

Statistical analysis of co-infection in *I. ricinus* revealed significant differences among infected ticks (*χ*^2^_1_ = 4.81; *P* = 0.028). *Babesia*-positive *I. ricinus* ticks were more frequently observed among *Borrelia*-positive ticks (2.7%; 8/290) than among ticks uninfected with *Borrelia* (0.8%; 7/810). The most frequent dual co-infections were between *B. afzelii* and *B. microti* (3/7), followed by *B. afzelii* and *B. venatorum* (2/7; Table 3).

## Discussion

We performed a long-term study of *Borrelia* and *Babesia* prevalence in two tick species removed from humans in Poland. The results of this study may have important implications in terms of public health since the prevalence of *B. burgdorferi* (*s.l.*) spirochetes in ticks has been considered an essential element of risk assessment for LB [35].

The ticks collected in this study belonged to two species*.* While almost the whole of Europe is considered to be an endemic region for *I. ricinus*, the geographical range of *D. reticulatus* in Europe is discontinuous, with two main macroregions, and the spreading of *D. reticulatus* is believed to be associated with the loss of forest area [36]. The adult ticks collected in our study appeared to show a bimodal activity pattern, with the highest density in March–May and September–November, whereas no ticks were collected in the summer, which is typical for this tick species [37]. *Dermacentor reticulatus* represents the second genus of medical and veterinary importance after *Ixodes* in Europe. This tick species can bite humans [38] and is sporadically removed from patients in Germany, Belgium and Poland [39–41]. Several pathogens, including *B. burgdorferi*, have been detected in *D. reticulatus* ticks, suggesting a possible role of this tick species in the life-cycle and transmission of spirochetes, but it does not necessarily mean that they are capable of transmitting these bacteria to a susceptible host. Previous studies have shown that *Borrelia* prevalence in questing *D. reticulatus* ticks is significantly lower (by up to 4%) [34, 37, 42, 43] than that reported in our study. Nevertheless, the infection rates reported in engorged *D. reticulatus* ticks collected from dogs in a previous study [27] are similar to our results.

For *I. ricinus,* we observed peak activity in June, which is congruent with the results of our previous study on questing ticks [24] as well as with the results of other studies on the seasonality of *I. ricinus* bites on humans [12, 13, 44, 45]. The predominance of nymphs according to the month of the year observed in our study was similar to that found in other European studies on ticks collected from humans [39–41, 46–48]. We found that the activity of larvae was the highest in August and September; however, only 54 specimens in total were removed from humans. It is worth noting that the highest number of tick bites occurred during the summer period when people are more likely to be exposed to ticks by spending time outdoors, not only in natural areas.

Overall in Europe, including Poland, *Borrelia* prevalence in ticks removed from humans ranges from 5 to 29% [39–41, 46–53]. Surprisingly, between 2016 and 2019, annual *Borrelia* prevalence in ticks decreased significantly from 38 to 25%. At the same time, the number of LB cases in Poland decreased slightly from 21,220 in 2016 to 20,614 in 2019 [2]. Our previous studies have shown that annual *Borrelia* occurrence in questing *I. ricinus* ticks in Poland also varied from 8 to 15% between 2013 and 2014 [24]. These inter-annual fluctuations in *Borrelia* prevalence may be due to climatic or other ecological factors affecting tick density or the abundance and, as a result, the availability of reservoir hosts, such as rodents or birds. It has been proven that the relative abundance of the white-footed mouse (*Peromyscus leucopus*) is positively associated with the prevalence of nymphal infection, which is regarded as the most important indicator of LB risk [54]. Interestingly, the *Borrelia* prevalence in our study differed significantly between *I. ricinus* ticks removed from humans and questing ticks [24]. Some results from other studies suggest that the abundance of spirochaetes in questing *Ixodes* ticks may be low (< 300 copies of bacteria) and, therefore, often undetectable, while blood repletion or simply the increased ambient temperature triggers bacteria growth and increases detectability, but possibly only within a short period (around 72 h after changing the conditions) [55, 56]. Understanding of this phenomenon is limited at the present time.

The observed significant lower *Borrelia* infection rates in *I. ricinus* larvae compared to nymphs and in nymphs compared to adults is in accordance with previous studies on questing as well as engorged ticks [24, 25, 39, 41, 47]. Since each tick stadium has only one blood meal from different hosts and the probability of acquiring pathogens increase with every blood meal, the highest prevalence of infection is noted in adults ticks. It is believed that transovarial transmission of *B. burgdorferi* (s.l*.*) is rare or non-existent and seems not to be essential for the circulation of spirochetes in Europe [57]. However, van Duijvendijk et al. [58] showed that flagged larvae can transmit *B. afzelii* and *B. miyamotoi* to rodents. Compared to other *Borrelia* species, a significantly higher efficiency of transovarial transmission from infected females to 90% of their larvae has been noted for *B. miyamotoi* [59]*.* Thus, in our study, larvae infected mainly with *B. miyamotoi* were expected. However, detection of the spirochetes in larvae in both the present study and other studies [40, 48, 60] strengthens the body of evidence for transovarial transmission of *Borrelia* under field conditions. Nonetheless, Faulde et al. [50] did not confirm the case of acquired LB after the bite of an infected *I. ricinus* larva. Hence, the hypothesis of *Borrelia* transmission from larvae to human needs further experimental studies.

Since different *Borrelia* species are involved in specific clinical manifestations, it is crucial to have accurate numbers on the prevalence of a particular species in order to be able to make precise assessments. The identification of different *Borrelia* species in our study revealed that *B. afzelii* was the most frequent species within the study period; these results are comparable to data on questing and engorged ticks from other European countries (reviewed in [61]). The low frequency of *B. spielmanii* and *B. lusitaniae* could be explained by a relatively low abundance of the competent reservoir host for these species in Poland, mainly dormice (*Eliomys quercinus*,* Muscardinus avellanarius*) and lizards (*Lacerta agilis*) [62, 63]. Interestingly, in our study *I. ricinus* ticks removed from humans were more frequently infected with *B. miyamotoi* than was earlier reported for questing ticks [24], whereas the latter were significantly more often infected with *B. garinii*. We also observed that *B. afzelii* prevalence was higher in ticks removed from humans than in questing ticks. Similar results were obtained by Springer et al. [47] and Waindok et al. [39]. The differences in *Borrelia* prevalence in questing and engorged ticks might be the result of specific eco-epidemiological conditions within the habitats affecting the availability and abundance of reservoir hosts for ticks as well as for *Borrelia* spirochetes. Coipan et al. [64] also reported that *B. afzelii* and *B. bavariensis* were significantly more frequent in human cases than in questing ticks, which is related with the fact that both are rodent-associated *Borrelia* species. Rodents are the main reservoir hosts for these two *Borrelia* species, as well as for *I. ricinus* larvae and nymphs; therefore, this phenomenon might be the result of spatial overlap between habitats of rodents with areas of human activity where the risk of tick bites is significant [47, 64]. Nevertheless, no *B. bavariensis* isolates were observed in this study, likely due to using the single restriction enzyme* Dde*I, which is not able to distinguish the recently described *B. bavariensis* from *B. garinii* [21]*.* However, sequence analysis of *Borrelia* isolates collected in 2016 and 2017 did not confirm the presence of *B. bavariensis* species.

It is well-known that the distribution and prevalence of *Borrelia* spp. in questing and engorged ticks show significant temporal and spatial variations [10, 65] and that monitoring these changes might be an important indicator of risk assessment [66]. Surprisingly, in our study, the annual prevalence of *B. miyamotoi* was relatively high (up to 15.8% in 2016) compared to that reported in other European studies in questing as well as feeding ticks where the prevalence usually did not exceed 5–8% [24, 25, 47, 67–70]. Breuner et al. [70] showed that single *I. scapularis* nymphs effectively transmit *B. miyamotoi* while feeding and that transmission can occur within the first 24 h of nymphal attachment. Additionally, probably due to the overlap of endemic areas for *B. miyamotoi* with those for the *B. burgdorferi* (*s.l.*) complex, co-infections of *B. miyamotoi* with other spirochete species in *I. ricinus* ticks and humans have been observed [6, 24]. Taken together, these data indicate that the risk of *B. myiamotoi* infection in Poland should not be underestimated. So far, only one case of human *B. miyamotoi* infection has been diagnosed [71]. However, Fiecek et al. [71] suggested that in the case of the patients who do not meet the criteria for neuroboreliosis (presence of *B. burgdorferi* antibodies only in serum, no antibodies in polymyalgia rheumatica [PMR]), *B. miyamotoi* disease should be considered. According to the National Institute of Public Health–National Institute of Hygiene in Poland [2], in 2013 only 14% of all reported cases with neurological symptoms (*n* = 1267) met the clinical and laboratory criteria of neuroborreliosis (detection of antibodies in PMR) [71].

Co-infections in ticks are frequently reported, likely due to a large variety of animals from which they can ingest blood. Such co-infections may result in a combination of vertical (transovarial) and horizontal (blood meal) transmission of *Borrelia* species as well as a combination of systemic and co-feeding transmission [72]. Larval ticks taking multiple blood meals from different hosts (interrupted blood meals [59]) could also produce double infections of various host-specific species. The observed rate of co-infection prevalence in our study was significantly lower than that reported in feeding *I. ricinus* ticks in other European studies [39, 47]. Dual co-infection involving *B. afzelii*/*B. burgdorferi* or *B. afzelii*/*B. miyamotoi* were observed in nymphs, possibly a result of simultaneous infection during blood-feeding on rodents in the larval stadium. However, transovarial transmission of *B. miyamotoi* is also possible. Herrmann et al. [72] reported that *Borrelia* species which share the same vertebrate reservoir hosts frequently occur together and exhibit weak inhibition and even facilitation with respect to the spirochete load inside the nymphal tick. These authors also observed that rodents are relevant reservoir hosts for *B. miyamotoi* and *B. afzelii*, which explains the high number of double infections involving these two species. Alternatively, the co-occurrence of these two species in nymphs may be due to a combination of transovarial transmission of *B. miyamotoi* and horizontal transmission of *B. afzelii* [72].

Co-infections with *B. afzelii*/*B. spielmanii* and *B. afzelii*/*B. burgdorferii*/*B. lusitaniae* noted in *I. ricinus* females were likely the result of acquiring pathogens during successive blood meals on different reservoir hosts. Herrmann et al. [72] reported that *B. burgdorferi* (*s.l.*) species pairs that are specialized on different vertebrate reservoir hosts rarely occur together and that they exhibit strong inhibition with respect to spirochete load. This negative association between occurrence and spirochete load inhibition is likely caused by the vertebrate immune system (vertebrate complement system), which is present in the tick midgut and is capable of lysing spirochetes that are not adapted to that particular vertebrate host [72–74]. Nonetheless, the low prevalence of co-infection with different *Borrelia* species has suggested that the risk of this type co-infection in humans in Poland is low.

We found the prevalence of *Babesia* in the *I. ricinus* ticks removed from humans to be low and similar to that reported in other European studies on engorged as well as questing *I. ricinus* ticks [41, 75, 76]. In Europe, the majority of human babesiosis cases are caused by *Babesia divergens* [7]. However, in Poland so far only *B. microti* infections in humans have been noted [77–80]. Additionally, *B. microti* species occurred significantly more often than *B. divergens* in questing [81–83] and engorged (this study) *I. ricinus* ticks.

Recent studies concentrating on *Babesia microti* and *Borrelia burgdorferi* (*s.s.*) infections in rodents and ticks have indicated that co-infection with these pathogens is common in vectors and enzootic hosts, with a greater probability of co-infection than that predicted by chance, suggesting that co-infection provides a survival advantage to both pathogens; however, the molecular mechanism of these facilitation remain still unclear [18]. In our study, *Babesia*-positive *I. ricinus* ticks were observed significantly more often among *Borrelia*-positive ticks than among ticks not infected with *Borrelia*. However, it is difficult to conclude whether the observed findings are the result of positive interactions between these pathogens. Of the seven co-infections, three involved *B. afzelii* and *B. microti*, species that are associated with the same animal host (rodents); as such, the infections may have been acquired simultaneously, especially in nymphs. Diuk-Wasser et al. [18] discussed in their review that infection with Lyme spirochetes promotes the uptake of *B. microti* by ticks from the host (particularly reservoir host) and increases the suitability of the reservoir host for these parasites. Co-infections with other *Borrelia/Babesia* species in *I. ricinus* females are probably the consequences of sequential infection during feeding on various vertebrate hosts. Despite the fact that the *Borrelia/Babesia* species considered to be pathogenic for humans were involved in the majority of detected coinfections, its low prevalence suggests that the risk of this type of co-infection in humans in Poland is negligible.

## Conclusions

In conclusion, our study confirms a relatively high *Borrelia* prevalence in ticks removed from humans, with the spirochete species showing a significant annual variation. Although based on detected sequences, *B. afzelii* constitutes the majority pathogenic species, the risk of *B. miyamotoi* disease in humans should not be underestimated. Analysis of *Babesia* prevalence suggests that the risk of human babesiosis is negligible, which is consistent with the low number of babesiosis cases reported in Poland. Although the overall risk of developing LB after a tick bite in Europe is 4% [84], increased knowledge of the prevalence and distribution of *Borrelia* and *Babesia* species in ticks may be an important indicator of both tick-borne disease risk assessment and varying pathogenicity in humans.

## Supplementary Information

**Additional file 1**: The number of* I. ricinus* ticks by stage, month and year collected during four years of study.

**Additional file 2**: The number of* D.reticulatus* ticks by stage, month and year collected during the 4 years of the study

**Additional file 3**: *Borrelia* genospecies/species distribution in infected *I. ricinus* ticks (*n* = 251) removed from humans between 2016 and 2019.

**Additional file 4**: Stadium and year distribution of* Babesia*-infected* I. ricinus* ticks removed from humans in 2016, 2017 and 2018.

## Data Availability

The datasets generated during and/or analyzed during the current study are available from the corresponding author on reasonable request.
