# Supplementary material for: Long-term study of Borrelia and Babesia prevalence and co-infection in Ixodes ricinus and Dermacentor recticulatus ticks removed from humans in Poland, 2016–2019
Source: Parasit Vectors. 2021 Jul 1;14:348. doi: 10.1186/s13071-021-04849-5 (PMC8252237; doi:10.1186/s13071-021-04849-5)
Supplement: Supplementary file 2 — Additional file 2: The number of D.reticulatus ticks by stage, month and year collected during the 4 years of the study [file 13071_2021_4849_MOESM2_ESM.docx]

Additional file 2. The number of *D. reticulatus* ticks by stage, month and year collected during four years of study
