# Supplementary material for: Long-term study of Borrelia and Babesia prevalence and co-infection in Ixodes ricinus and Dermacentor recticulatus ticks removed from humans in Poland, 2016–2019
Source: Parasit Vectors. 2021 Jul 1;14:348. doi: 10.1186/s13071-021-04849-5 (PMC8252237; doi:10.1186/s13071-021-04849-5)
Supplement: Supplementary file 3 — Additional file 3: Borrelia genospecies/species distribution in infected I. ricinus ticks (n = 251) removed from humans between 2016 and 2019. [file 13071_2021_4849_MOESM3_ESM.docx]

|  | | No of infected *I. ricinus* ticks (%; 95% confidence interval) | | | | | | | |
| --- | --- | --- | --- | --- | --- | --- | --- | --- | --- |
|  |  | No of tested ticks (No of positive) | *B. afzelii* | *B. garinii* | *B. burgdorferi* | *B. miyamotoi* | *B. valaisiana* | *B. lusitaniae* | *B. spielmanii* |
| Total | | **251** | **164 (65.3; 59.3-71.0)** | **22 (8.8; 5.7-12.7)** | **27 (10.8; 7.4-15.0)** | **21 (8.4; 5.4-12.3)** | **13 (5.2; 2.9-8.4)** | **1 (0.4; 0.0-1.8)** | **3 (1.2; 0.3-3.2)** |
| Year of study | 2016 | 38 (38) | 23 (60.5; 44.7-74.8) | 3 (7.9; 2.3-19.6) | 3 (7.9; 2.3-19.6) | 6 (15.8; 6.9-29.7) | 2 (5.3; 1.1-15.8) | 1 (2.6; 0.3-11.6) | 0 |
|  | 2017 | 64 (66) | 39 (60.9; 48.7-72.2) | 15 (23.4; 14.4-34.8) | 2 (3.1; 0.7-9.6) | 2 (3.1; 0.7-9.6) | 6 (9.4; 4.0-18.3) | 0 | 0 |
|  | 2018 | 77 (194) | 60 (77.9; 67.7-86.1) | 1 (1.3; 0.1-5.9) | 7 (9.1; 4.2-17.0) | 5 (6.5; 2.5-13.6) | 3 (3.9; 1.1-10.0) | 0 | 1 (1.3; 0.0-5.9) |
|  | 2019 | 72 (181) | 42 (58.3; 46.8-69.2) | 3 (4.2; 1.2-10.7) | 15 (20.8; 12.7-31.2) | 8 (11.1; 5.4-19.9) | 2 (2.8; 0.6-8.6) | 0 | 2 (2.8; 0.6-8.6) |

Additional file 3. *Borrelia* genospecies/species distribution in infected *I. ricinus* ticks (n = 251) removed from humans between 2016 and 2019
