# Supplementary material for: Long-term study of Borrelia and Babesia prevalence and co-infection in Ixodes ricinus and Dermacentor recticulatus ticks removed from humans in Poland, 2016–2019
Source: Parasit Vectors. 2021 Jul 1;14:348. doi: 10.1186/s13071-021-04849-5 (PMC8252237; doi:10.1186/s13071-021-04849-5)
Supplement: Supplementary file 4 — Additional file 4: Stadium and year distribution of Babesia-infected I. ricinus ticks removed from humans in 2016, 2017 and 2018. [file 13071_2021_4849_MOESM4_ESM.docx]

Additional file 4. Stadium and year distribution of *Babesia*-infected *Ixodes ricinus* ticks removed from humans in 2016 and 2019

|  | No. of tested  ticks | *Babesia*-positive *I. ricinus* ticks  No of positive ticks (%; 95% confidence interval) | | | |
| --- | --- | --- | --- | --- | --- |
|  |  | 2016 | 2017 | 2018 | Total |
| Larvae | 41 | 0 | 0 | 0 | 0 |
| Nymphs | 738 | 2 (2.5; 0.5-7.8) | 1 (0.8; 0.1-3.5) | 4 (0.8; 0.3-1.8) | 7 (0.9; 0.4-1.9) |
| Adults | 336 | 1 (2.6; 0.3-11.4) | 1 (1.2; 0.1-5.3) | 6 (2.8; 1.2-5.7) | 8 (2.4; 1.1-4.4) |
| **Total** | **1115** | **3 (2.4; 0.7-6.2)** | **2 (0.9; 0.2-2.8)** | **10 (1.3; 0.7-2.3)** | **15 (1.3; 0.8-2.2)** |
